# Integration of a novel anti-PD-1 antibody with chimeric antigen receptor-T engineered to express interleukin-7 enhances targeting efficacy against lung cancer

**DOI:** 10.1093/lifemedi/lnaf035

**Published:** 2025-12-23

**Authors:** Chenxi Cheng, Lin Zhang, Jiani Cao, Xiaoyan Li, Ya Wen, Kun Liu, Tongbiao Zhao

**Affiliations:** Key Laboratory of Organ Regeneration and Reconstruction, State Key Laboratory of Stem Cell and Reproductive Biology, Institute for Stem Cell and Regeneration, Institute of Zoology, Chinese Academy of Sciences, Beijing 100101, China; University of Chinese Academy of Sciences, Beijing 100049, China; Beijing Institute for Stem Cell and Regenerative Medicine, Beijing 100101, China; Key Laboratory of Organ Regeneration and Reconstruction, State Key Laboratory of Stem Cell and Reproductive Biology, Institute for Stem Cell and Regeneration, Institute of Zoology, Chinese Academy of Sciences, Beijing 100101, China; University of Chinese Academy of Sciences, Beijing 100049, China; Beijing Institute for Stem Cell and Regenerative Medicine, Beijing 100101, China; Key Laboratory of Organ Regeneration and Reconstruction, State Key Laboratory of Stem Cell and Reproductive Biology, Institute for Stem Cell and Regeneration, Institute of Zoology, Chinese Academy of Sciences, Beijing 100101, China; University of Chinese Academy of Sciences, Beijing 100049, China; Key Laboratory of Organ Regeneration and Reconstruction, State Key Laboratory of Stem Cell and Reproductive Biology, Institute for Stem Cell and Regeneration, Institute of Zoology, Chinese Academy of Sciences, Beijing 100101, China; University of Chinese Academy of Sciences, Beijing 100049, China; Key Laboratory of Organ Regeneration and Reconstruction, State Key Laboratory of Stem Cell and Reproductive Biology, Institute for Stem Cell and Regeneration, Institute of Zoology, Chinese Academy of Sciences, Beijing 100101, China; University of Chinese Academy of Sciences, Beijing 100049, China; Beijing Institute for Stem Cell and Regenerative Medicine, Beijing 100101, China; Key Laboratory of Organ Regeneration and Reconstruction, State Key Laboratory of Stem Cell and Reproductive Biology, Institute for Stem Cell and Regeneration, Institute of Zoology, Chinese Academy of Sciences, Beijing 100101, China; University of Chinese Academy of Sciences, Beijing 100049, China; Beijing Institute for Stem Cell and Regenerative Medicine, Beijing 100101, China; Key Laboratory of Organ Regeneration and Reconstruction, State Key Laboratory of Stem Cell and Reproductive Biology, Institute for Stem Cell and Regeneration, Institute of Zoology, Chinese Academy of Sciences, Beijing 100101, China; University of Chinese Academy of Sciences, Beijing 100049, China; Beijing Institute for Stem Cell and Regenerative Medicine, Beijing 100101, China

**Keywords:** chimeric antigen receptor T (CAR-T), programmed cell death protein 1 (PD-1), monoclonal antibody (MAb), interleukin-7 (IL-7)

## Abstract

Chimeric antigen receptor (CAR) T cell therapy has emerged as a promising approach for hematological malignancies, yet its efficacy in solid tumors is hindered by limited persistence. To address this, immune checkpoint inhibitors (ICIs) and cytokines have been explored as potential solutions. In this study, we developed a novel monoclonal antibody (mAb), m8A8, which exhibits high specificity for human PD-1 and effectively disrupts its ligand interactions. Furthermore, we engineered CAR-T cells to express human IL-7, resulting in enhanced anti-tumor efficacy in xenograft models. Additionally, the human–mouse chimeric antibody C8A8, derived from m8A8, was found to significantly amplify the anti-tumor activity of IL-7-engineered CAR-T cells. Our findings provide compelling evidence and a robust rationale for the synergistic integration of ICIs, cytokines, and CAR-T cell therapy in the treatment of solid tumors.

## Introduction

Immunotherapy represents a transformative approach in the fight against cancer, offering potential solutions to the limitations of conventional treatments. Immune checkpoint inhibitors (ICIs) have demonstrated remarkable efficacy in tumors characterized by high lymphocyte infiltration. However, their effectiveness is significantly reduced in patients with immunologically “cold” tumors, which often do not respond to these agents [1, 2]. Chimeric antigen receptor T (CAR-T) cell therapy has emerged as a groundbreaking advancement, showing substantial success, particularly in hematologic malignancies. Yet, its application to solid tumors presents notable challenges [3–5].

In solid tumors, T cells often become exhausted and express various inhibitory receptors on their surface, including programmed cell death protein 1 (PD-1), cytotoxic T-lymphocyte-associated antigen 4 (CTLA-4), T-cell immunoglobulin and mucin-domain containing-3 (TIM-3), and lymphocyte-activation gene 3 (LAG3) [6–9]. Recently, ICIs targeting the PD-1/PD-L1 pathway have been employed in treating a range of solid tumors [10–12]. Despite their success, these inhibitors have shown significant therapeutic effects primarily in specific tumor types, such as non-small cell lung cancer (NSCLC). Nivolumab (Nivo), an anti-PD-1 antibody, has demonstrated considerable promise in NSCLC treatment by blocking the interaction of PD-1 with PD-L1 or PD-L2 [13]. However, despite the promising preclinical results, only a minority of patients respond positively to monotherapy, posing a significant challenge in NSCLC treatments [14, 15]. This underscores the urgent need for novel antibody drugs and combination therapies to overcome resistance to anti-PD-1 antibodies.

The limited persistence of CAR-T cells in solid tumors is often due to reduced self-renewal rates and exhaustion. Interleukin-7 (IL-7) has been identified as a potent enhancer of T-cell proliferation and maturation, promoting the expansion of stem central memory (SCM) and central memory (CM) T cell subsets, thereby enhancing anti-tumor activity [16–19]. Building on our previous research, which demonstrated the efficacy of preconditioning CAR-T cells with IL-7/IL-15 to augment their anti-tumor capabilities [20], recent studies have shown promise in combining anti-PD-1 antibodies with CAR-T cell therapy for cancer treatment [21–23]. Therefore, it is crucial to develop CAR-T cell products capable of generating sustained anti-tumor responses while effectively synergizing with anti-PD-1 therapy.

In this study, we developed monoclonal antibodies (mAbs) against PD-1 using hybridoma technology. Among them, m8A8 exhibited specific recognition of human PD-1 and effectively inhibited the PD-1/PD-L1 interaction. The human/murine chimeric antibody C8A8, derived from m8A8, successfully blocked the interaction between PD-1 and its ligands, PD-L1 and PD-L2. Additionally, we engineered CAR-T cells with IL-7, resulting in enhanced proliferation capacity and potent anti-tumor activity. Furthermore, the combination of C8A8 with IL-7-CAR-T cells significantly increased IL-2 and IFN-γ secretion and demonstrated strong anti-tumor activity. Notably, C8A8 showed comparable biological activity to Nivo. In summary, our study provides compelling evidence supporting the enhancement of CAR-T cell therapy for NSCLC by combining IL-7 and anti-PD-1 antibody. This approach holds promise for advancing cancer immunotherapy, particularly when utilized in conjunction with adjuvant immunotherapy strategies.

## Results

### Generation of anti-PD-1 monoclonal antibodies

**Figure 1.**
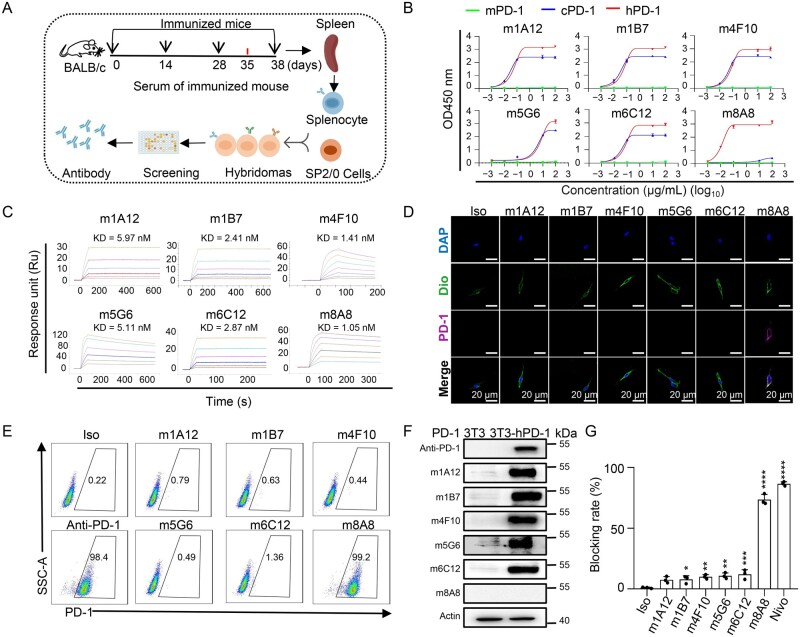
**Generation of anti-PD-1 monoclonal antibody targeting surface PD-1**.(A) Schematic representation of the workflow for producing anti-PD-1 mAbs using the hybridoma technique. (B) The specificity of the anti-PD-1 antibodies was assessed by ELISA, revealing that all anti-PD-1 antibodies could bind to human PD-1 protein but not to mouse PD-1 protein, with the m8A8 mAb showing exclusive binding to human PD-1 protein while the m1A12, m1B7, m4F10, m5G6 and m6C12 binding to cynomolgus monkey PD-1 proteins. (C) SPR analysis demonstrated the binding of anti-PD-1 antibodies to hPD-1 protein. (D) PD-1 immunofluorescence staining of 3T3-hPD-1 cells using six of the generated antibodies (m1A12, m1B7, m4F10, m5G6, m6C12, m8A8) indicated that only m8A8 specifically bound to the PD-1 protein on the membranes of the 3T3-hPD-1 cells. (E) Flow cytometry analysis showed that m8A8 specifically recognized 3T3-hPD-1 cells, while the other five mAbs did not bind to these cells. (F) Immunoblotting of PD-1 demonstrated antigen recognition by the six mAb clones and a commercially available anti-PD-1 mAb, which served as a control. (G) Competition ELISA assays were conducted to determine the blocking effect of the six anti-PD-1 antibodies on the PD-1/PD-L1 interaction, with m8A8 mAb exhibiting effective inhibition compared to the other anti-PD-1 antibodies, which showed less efficacy. Data are presented as the mean ± SD, *n* = 3; *P < 0.05, **P < 0.01, ***P < 0.001, ****P < 0.0001, Student’s *t*-test.

To generate monoclonal antibodies targeting PD-1, mice were immunized with two distinct immunogens: human PD-1 protein peptides (ex-PD-1 protein, residues F24-V170, UniProt: Q15116) and cells overexpressing human PD-1 (residues M1-L288, including signal peptide). Monoclonal antibodies were subsequently produced using hybridoma technology, as outlined in Fig. 1A. The ex-PD-1 protein immunogen was generated using an insect expression system, and the cell immunogen was established through stable overexpression of human PD-1 protein on the surface of 3T3 cells and A549 cells (Fig. S1A and S1B). Following immunization with these two immunogens, we assessed antibody secretion in the serum individually (Fig. S1C and S1D). Spleen cells from the immunized mice were then isolated to establish hybridoma cell lines capable of antibody secretion. Eventually, m1A12, m1B7, m4F10, m5G6, and m6C12, derived from ex-PD-1 protein immunization, and m8A8, derived from cell immunization, were obtained with binding activities to PD-1 proteins (Fig. 1B).

We next evaluated the specificity of the anti-PD-1 monoclonal antibodies. All antibodies specifically recognized human P D-1 without cross-reactivity towards murine PD-1 (Fig. 1B). Remarkably, except for m8A8, the other five antibodies also recognized cynomolgus monkey PD-1 (Fig. 1B). Subsequently, we determined the affinity of these antibodies using surface plasmon resonance (SPR) measurements. The results revealed that all anti-PD-1 antibodies bound to human PD-1 with dissociation constant (KD) values of 5.97, 2.41, 1.41, 5.11, 2.87, and 1.05 nM, respectively (Fig. 1C). Immunofluorescence staining and flow cytometry demonstrated that m8A8 could recognize 3T3-hPD-1 cells, whereas the other five antibodies could not bind to these cells (Fig. 1D and 1E). Western blot analysis revealed that only m8A8 among these 6 antibodies failed to recognize fully denatured hPD-1 (Fig. 1F). Competitive ELISA experiments showed that only m8A8 effectively blocked the interaction between PD-1 and PD-L1, while the other five antibodies exhibited limited blocking efficacy (Fig. 1G).

### Development of human-mouse chimeric antibody C8A8

**Figure 2.**
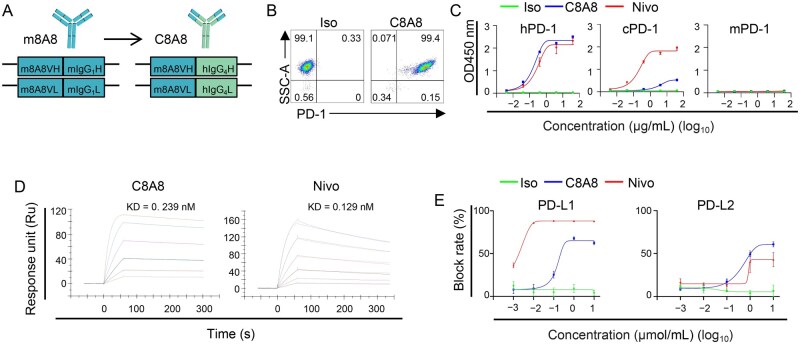
**Development of the human–mouse chimeric antibody C8A8**.(A) Schematic diagram illustrating the structure of the C8A8 antibody. (B) Assessment of C8A8 mAb binding to 3T3-hPD-1 cells using flow cytometry, with an isotype control (Iso) serving as a negative control. (C) Binding assessment of C8A8 to recombinant human, cynomolgus monkey, and mouse PD-1 proteins by ELISA, demonstrating that C8A8 specifically recognized human PD-1 protein but did not bind to cynomolgus monkey or mouse PD-1 protein. (D) Kinetic measurements of C8A8 antibody binding to recombinant human PD-1 using SPR, with Nivo serving as a positive control. (E) Competition ELISA showing the binding inhibition of hPD-1 and PD-L1/PD-L2 by C8A8, with Nivo as a positive control and the Iso as a negative control.

To advance the clinical application of anti-PD-1 antibodies in immunotherapy, we engineered the monoclonal antibody m8A8 with human IgG4, resulting in the human–mouse chimeric antibody C8A8 (Fig. 2A). C8A8 demonstrated specific recognition of human PD-1, without cross-reactivity observed with PD-1 protein from cynomolgus monkey or mice (Fig. 2B and 2C). Notably, C8A8 exhibited a high binding affinity to human PD-1 as well, with a KD of 0.239 nM, similar to Nivo, which binds with a KD of 0.129 nM (Fig. 2D). The blocking efficacy of C8A8 was evaluated through ELISA-based assays, confirming its ability to effectively inhibit the interaction between PD-1 and its ligands, PD-L1 or PD-L2, similar to Nivo (Fig. 2E).

### Engineering IL-7 in CAR-T cells enhances their anti-tumor activity

**Figure 3.**
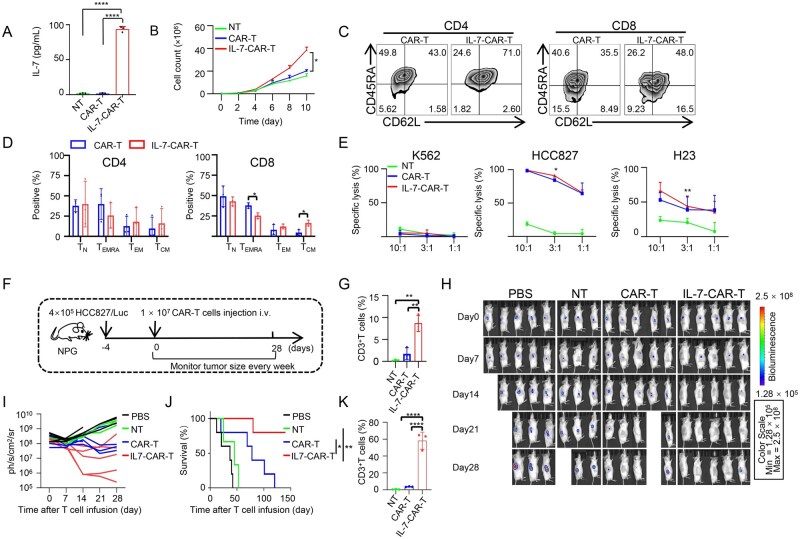
**Engineering IL-7 in CAR-T cells enhances their anti-tumor activity**.(A) Measurement of IL-7 secretion in culture supernatant by ELISA. Data are presented as the mean ± SD, *n* = 3, ****P < 0.0001. (B) Assessment of proliferation capacity of IL-7-CAR-T cells by cell count. NT, CAR-T, and IL-7-CAR-T cells were cultured with an initial cell count of 2 × 10^5^, and cell numbers were counted every other day. Data shown are the mean ± SD, *n* = 3; *P < 0.05, Student’s *t*-test. (C) The expression of CD45RA and CD62L on CAR-T cells and IL-7-CAR-T cells were analyzed by FACS. (D) Statistical analysis of memory T cell phenotypes. Data are shown as the mean ± SD, *n* = 3, *P < 0.05, Student’s *t*-test. (E) Evaluation of specific cytotoxicity of IL-7-CAR-T cells against lung cancer cell lines (HCC827, H23) using luciferase-based assays at various effector-to-target (E: T) ratios (10:1, 3:1, and 1: 1), with K562 cells as a negative control. Data are presented as the mean ± SD, *n* = 3, *P < 0.05, **P < 0.01, Student’s *t*-test. (F) The experimental timeline for the treatment of cell line-derived xenograft (CDX) tumor model. 4 × 10^5^ HCC827/Luc cells were subcutaneously injected into NPG mice. After 4 days, tumor-bearing NPG mice were treated intravenously with PBS, 1 × 10^7^ NT, CAR-T, and IL-7-CAR-T cells. (G) Peripheral blood T-cell counts were quantified by flow cytometry at 10 days post-adoptive transfer. Data are shown as the mean ± SD, *n* = 3; **P < 0.01, Student’s *t*-test. (H) Evaluation of the anti-tumor activity of IL-7-CAR-T cells by *in vivo* imaging in mice bearing HCC827 xenografts. Tumor growth was monitored over 28 days. (I) Bioluminescence signals from each mouse, as in (H), were recorded every 7 days. (J) The survival of tumor-bearing mice treated with PBS, NT, CAR-T cells and IL-7-CAR-T cells. PBS, CAR-T cells and IL-7-CAR-T cells group *n* = 5, NT group *n* = 3, *P < 0.05, **P < 0.01, log-rank test. (K) Tumor-infiltrating T cell populations were quantified by flow cytometry at 10 days post-adoptive transfer. Data are shown as the mean ± SD, *n* = 3; ****P < 0.0001, Student’s *t*-test.

As EGFR was observed to be highly expressed in some NSCLC cell lines [24], we evaluated the expression of EGFR and PD-L1 in lung cancer cell lines HCC827 and H23, with K562 cells as a control. The experimental results demonstrated high expression levels of both PD-L1 and EGFR in these cell lines (Fig. S2A and S2B). Building on this, we engineered CAR-T cells targeting EGFR. To boost the anti-tumor activity of these cells, we designed a second-generation CAR structure targeting EGFR that incorporates the full human IL-7 sequence (Fig. S3A). Human T cells were activated and transduced to express either CAR or IL-7-CAR, resulting in CAR-T or IL-7-CAR-T cells. After a 72-h incubation, flow cytometry was used to assess the proportion of CAR-positive cells, and ELISA measured IL-7 secretion in the supernatants. The results showed that IL-7-CAR-T cells not only expressed CAR but also efficiently secreted human IL-7 (Figs. 3A, S3B and S3C). IL-7 significantly enhanced the proliferation and inhibited apoptosis of CAR-T cells *in vitro* (Figs. 3B, S3D and S3E). To further elucidate the immunomodulatory effects of IL-7 on CAR-T cell differentiation, we performed phenotypic analysis based on the expression of CD45RA and CD62L. Compared to conventional CAR-T cells, IL-7-engineered CAR-T cells exhibited a significantly higher frequency of central memory-like CD8^+^ T cells (T_CM_, CD45RA^−^CD62L^+^), indicating a shift toward a less differentiated, long-lived phenotype (Fig. 3C and 3D). Moreover, IL-7 expression was associated with a marked reduction in the expression of key exhaustion markers, including PD-1, Tim3 and LAG-3, further supporting its role in preserving CAR-T cell functional persistence (Fig. S3F).

To assess the cytotoxicity of IL-7-CAR-T cells against tumor cells, we conducted a luminescent assay at various effector-to-target (*E*:*T*) ratios. The data indicated that IL-7 supplementation significantly enhanced the cytotoxicity of CAR-T cells *in vitro* (Fig. 3E). Furthermore, to evaluate the *in vivo* anti-tumor activity of IL-7-CAR-T cells, we generated xenograft tumor-bearing mice by subcutaneously injecting HCC827/Luc cells into 7-week-old male NPG mice. Four days later, tumor-bearing mice received intravenous injections of either 1 × 10^7^ CAR-T or IL-7-CAR-T cells (Fig. 3F). Tumor size was monitored every 7 days. Peripheral blood analysis at day 10 post-infusion revealed a markedly increased frequency of circulating T cells in mice receiving IL-7-CAR-T cells, indicating that IL-7 expression promotes robust *in vivo* T cell expansion and persistence (Fig. 3G). Both CAR-T and IL-7-CAR-T cells groups exhibited significantly smaller tumor sizes compared to non-treated (NT) groups on day 14. Notably, IL-7-CAR-T cells were able to clear the tumor in 14 days, which was faster than the CAR-T cells (Fig. 3H and 3I). Additionally, IL-7-CAR-T cells significantly prolonged the survival of the tumor-bearing mice compared to CAR-T cells (Fig. 3J). To assess the proliferative capacity of IL-7-CAR-T cells within the tumor microenvironment, we injected 2.5 × 10^6^ cells (NT, CAR-T, or IL-7-CAR-T) into pre-established tumor sites using intratumoral injection. Ten days post-treatment, tumor tissues were harvested for flow cytometric analysis of tumor-infiltrating T lymphocytes. Compared to NT and conventional CAR-T cells, the IL-7-CAR-T cells exhibited significantly greater T-cell expansion within the tumor, indicating enhanced local persistence and proliferation (Fig. 3K). These findings collectively demonstrate that IL-7 expression augments CAR-T cell accumulation at tumor sites and enhances anti-tumor efficacy in solid tumors.

To evaluate potential IL-7-associated toxicity, major organs—including the heart, liver, spleen, lung, kidney, and ovary—were collected from NPG mice 10 days post-infusion for histopathological examination using hematoxylin and eosin (H&E) staining and CD3 immunohistochemistry (IHC). H&E staining revealed no histological abnormalities or tissue injury in any of the organs examined, suggesting the absence of IL-7-mediated systemic toxicity (Fig. S4A). CD3 IHC staining confirmed that IL-7-CAR-T cells were predominantly localized within lymphoid tissue (spleen), with no detectable infiltration into non-lymphoid organs (Fig. S4B). Together, these results demonstrate that IL-7-CAR-T cell therapy does not elicit significant off-target tissue damage, supporting its favorable safety profile.

### Enhancing immunotherapy against lung cancer by integration of IL-7-CAR-T with anti-PD-1 antibody

**Figure 4.**
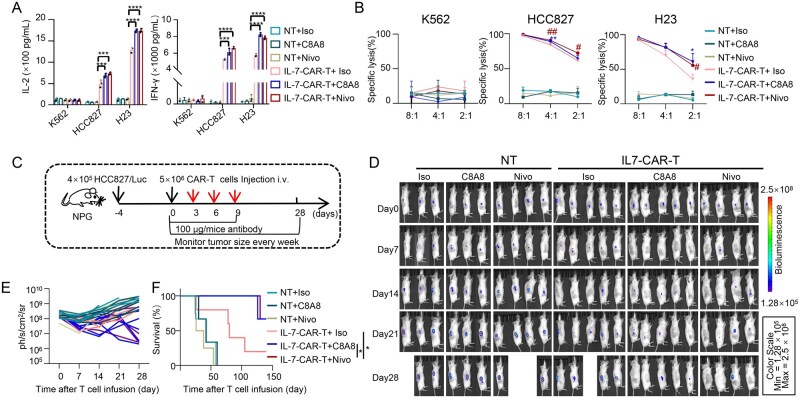
**Integration of IL-7-CAR-T with anti-PD-1 antibody significantly improves immunotherapy against lung cancer**. (A) Detection of IL-2 and IFN-γ secretion in IL-7-CAR-T cells after co-culture with tumor cells (HCC827, H23, and K562) at an *E*:*T* ratio of 4:1, with 30 μg/mL C8A8. Iso and Nivo served as negative and positive controls, respectively. Data are shown as the mean ± SD, *n* = 3; ***P < 0.001, ****P < 0.0001, Student’s *t*-test. (B) C8A8 enhances the anti-tumor activity of IL-7-CAR-T cells *in vitro*. IL-7-CAR-T cells were co-incubated with HCC827, H23, and K562 cells at varying E: T ratios for 18 h with the presence of Iso, C8A8, and Nivo, respectively. Specific cytotoxicity was measured by Luciferase-based assays. Data are shown as the mean ± SD, *n* = 3; *P < 0.05, **P < 0.01, ^#^*P* < 0.05, ^##^*P* < 0.01, Student’s *t*-test. (C) Experimental timeline for the treatment of CDX tumor model using anti-PD-1 antibody and IL-7-CAR-T cells. NPG mice were injected subcutaneously with 4 × 10^5^ HCC827/Luc cells. After 4 days, mice bearing tumors were intravenously infused with 5 × 10^6^ NT and IL-7-CAR-T cells, respectively. The mice were intravenously injected with C8A8 (5 mg/kg, *n* = 5) or Nivo (5 mg/kg, *n* = 5) or isotype control antibody (5 mg/kg, *n* = 5) on day 0, 3, 6, and 9. (D) Representative images of tumor regression in tumor-bearing mice treated. Monitoring of tumor growth over 28 days. (E) Bioluminescence signals of each mouse from (D) were recorded at the indicated time points. (F) Combination therapy of IL-7-CAR-T cells with anti-PD-1 antibodies significantly prolongs the survival of tumor-bearing mice compared to the control groups. Data are shown as the mean ± SD; *P < 0.05, log-rank test.

To assess the potential of C8A8 in enhancing the anti-tumor activity of IL-7-CAR-T cells, we conducted co-culture experiments with various tumor cell lines, including HCC827, H23, and K562, at different effector-to-target (*E*:*T*) ratios in the presence of 30 µg/mL C8A8. Our data revealed that IL-7-CAR-T cells secreted significantly higher levels of IL-2 and IFN-γ when co-cultured with HCC827 and H23 cells in the presence of either C8A8 or Nivo, compared to the isotype control (Iso) (Fig. 4A). In contrast, no such cytokine secretion was observed when IL-7-CAR-T cells were co-cultured with K562 cells, regardless of the presence of C8A8 or Nivo. These results suggest that the anti-PD-1 antibody can potentiate the activation of IL-7-CAR-T cells. Moreover, both C8A8 and Nivo significantly enhanced the cytotoxicity of IL-7-CAR-T cells against HCC827 and H23 cells at E: T ratios of 4:1 and 2:1, compared to Iso (Fig. 4B).

To further evaluate the therapeutic efficacy of combining IL-7-CAR-T cells with C8A8 *in vivo*, we established a xenograft mouse model by subcutaneously injecting 4 × 10^5^ HCC827/Luc cells into the right flank of NPG mice. Following tumor establishment, NT, IL-7-CAR-T cells, and anti-PD-1 antibodies were administered intravenously to the tumor-bearing mice, with tumor progression monitored at various time points (Fig. 4C). The combination of IL-7-CAR-T cells with either C8A8 or Nivo resulted in a significant reduction in tumor growth since day 21, compared to the control group (Fig. 4D and 4E). Additionally, this combination therapy extended the survival of the tumor-bearing mice, demonstrating superior anti-tumor efficacy *in vivo* compared to controls (Fig. 4F). To comprehensively evaluate the safety of the combined IL-7-CAR-T and C8A8 antibody therapy, histopathological examination of major organs (heart, liver, spleen, lung, kidney, and ovary) was performed 10 days post-treatment. H&E staining revealed no evidence of tissue injury or inflammatory infiltration in any of the examined organs, indicating minimal off-target toxicity associated with the combination regimen (Fig. S5A). Subsequently, we evaluated the pharmacokinetic profile of the C8A8 antibody. ELISA-based quantification demonstrated that C8A8 exhibited a significantly extended serum half-life (*t*_1/2_ = 192 ± 12 h) compared to the clinically approved anti-PD-1 antibody Nivo (*t*_1/2_ = 168 ± 12 h), suggesting improved *in vivo* stability and sustained therapeutic potential (Fig. S5B). Collectively, these results underscore the favorable safety profile and enhanced pharmacological characteristics of IL-7-CAR-T cells when administered in combination with C8A8, supporting the translational potential of this strategy for solid tumor immunotherapy.

## Discussion

ICIs have transformed cancer treatment, offering substantial clinical benefits and durable responses, instilling hope for potential cures in more lung cancer patients [25]. Anti-PD-1 antibodies have been shown to significantly improve survival in NSCLC patients with PD-L1 expression on at least 50% of tumor cells [26]. In our study, we developed a novel monoclonal anti-PD-1 antibody, C8A8, which exhibits high affinity for PD-1 and effectively blocks the PD-1/PD-L1 interaction. Remarkably, C8A8 demonstrated comparable biological functionality to existing commercially antibodies. The design of C8A8 was based on m8A8, derived from immunization with 3T3-hPD-1 cells, which showed potent inhibition of the PD-1/PD-L1 interaction. Interestingly, anti-PD-1 antibodies generated from ex-PD-1 protein immunization exhibited limited efficacy in blocking this interaction. Western blot analysis demonstrated that m8A8 failed to bind denatured PD-1 protein, whereas both flow cytometry and immunofluorescence assays confirmed its specific recognition of native, cell-surface PD-1. These findings suggest that m8A8 recognizes a conformational epitope on PD-1, in contrast to conventional antibodies that target linear epitopes (Fig. 1D–F). These results indicate that m8A8 recognizes a conformational (non-linear) epitope on PD-1, distinguishing it from conventional anti-PD-1 antibodies that typically target linear epitopes exposed under denaturing conditions.

IL-7 is a pivotal homeostatic cytokine that plays an essential role in the development, survival, and functional maintenance of multiple lymphocyte lineages, including B cells, NK cells, and T cells. Early studies demonstrated that IL-7 significantly promotes B cell survival and enhances antibody production by supporting plasma cell differentiation and longevity [27, 28]. In the innate immune compartment, IL-7 has been shown to drive the expansion of NK cell populations and to potentiate their cytotoxic effector functions [29, 30]. Importantly, IL-7 is indispensable for the formation and long-term maintenance of immunological memory, as it governs the homeostasis and survival of both CD4^+^ and CD8^+^ memory T cell subsets through dynamic regulation of anti-apoptotic and proliferative signaling pathways. Previous research has highlighted the role of CAR-T cells with memory phenotypes in enhancing anti-tumor effects. Our previous studies have shown that the addition of IL-7 and IL-15 significantly improves the anti-tumor effect of CAR-T cells [31–35]. Clinical studies have demonstrated that IL-7 plays a pivotal role in enhancing the proliferation, survival, and functional competence of both CD4^+^ helper T cells and CD8^+^ cytotoxic T lymphocytes—key effector subsets that orchestrate adaptive anti-tumor immune responses [36]. Other studies have also demonstrated that the co-expression of IL-7 with CCL21 or CCL9 on CAR-T cells boosts their anti-tumor capabilities [37, 38]. Compared to conventional CAR-T cells, cytokine overexpression in CAR-T cells can lead to increased proliferation, persistence, and anti-tumor activity [39, 40]. Therefore, we directly integrated the complete human IL-7 sequenced into a second-generation CAR structure for use in CAR-T cell therapy. Persistent antigen stimulation can up-regulate immunosuppressive receptors, such as PD-1, in CAR-T cells, leading to treatment failure [41]. Some studies suggest that IL-7 can reduce the expression of inhibitory receptors on CAR-T cells [42, 43]. By incorporating IL-7, the CAR-T cells exhibited enhanced proliferation and persistence, thereby improving their anti-tumor activity. Furthermore, engineering CAR-T cells to express membrane-bound IL-7 has emerged as a promising strategy to enhance their *in vivo* persistence and anti-tumor efficacy. This approach has been shown to reduce relapse rates in patients with CD19^+^ B cell malignancies by promoting sustained T cell survival and functional fitness within the immunosuppressive tumor microenvironment [44].

**Figure 5.**
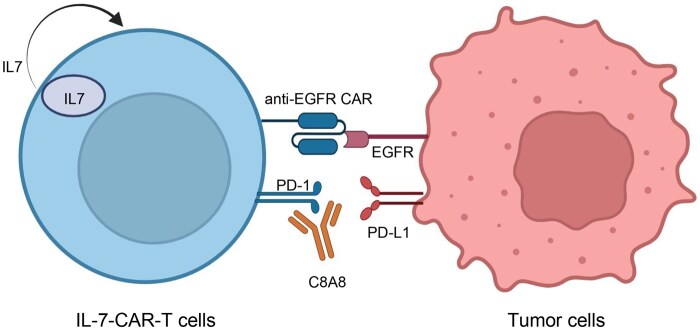
Schematic of the IL-7-CAR-T and anti-PD-1 antibody significantly improves immunotherapy against EGFR and PD-L1 positive cells.

While ICI monotherapy shows limited response in solid tumors, combining different ICIs with CAR-T cell therapy has emerged as a strategy to improve treatment efficacy [23, 45, 46]. Previous studies have shown promising results with combination therapies. For instance, the combined treatment of NSCLC with CAR-T cells targeting both MUC1 and PSCA antigens, along with anti-PD-1 antibodies, effectively enhances the killing capacity of CAR-T cells [47]. Other tumor model studies have demonstrated that a metastatic melanoma patient treated with a combination of nivolumab and ipilimumab initially showed no response but experienced tumor shrinkage upon retreatment with NT-I7 combined with pembrolizumab [48]. Additionally, Cherkassky previously demonstrated the synergistic effects of combining anti-MSLN-CAR-T cells with anti-PD-1 antibody, leading to enhanced CAR-T cell anti-tumor ability, which holds promise for extending patient survival [49]. In our study, the combination of anti-PD-1 antibody with IL-7-CAR-T cells effectively enhanced the tumor-killing efficacy of CAR-T cells. Our results suggest the potential of C8A8 in cancer immunotherapy, particularly in NSCLC when combined with IL-7-CAR-T cell therapy. IL-7 promotes T cell proliferation and activation, enhancing immune responses, while the anti-PD-1 antibody alleviates the negative regulatory effects of PD-1 signaling on CAR-T cells (Fig. 5). Consequently, the combination of IL-7 and C8A8 can synergistically enhance the anti-tumor activity of CAR-T cells, improving therapeutic efficacy.

## Research limitations

While the C8A8 antibody combined with IL-7-CAR-T cell therapy has shown promising anti-tumor activity in NSCLC treatment, further optimization is needed to improve therapeutic efficacy. In this study, IL-7 overexpression in CAR-T cells enhanced their proliferation and cytotoxicity. However, the fibrous stroma and extracellular matrix of solid tumors limit CAR-T cell infiltration and effectiveness. Future research should focus on optimizing CAR-T cells or therapeutic strategies by incorporating chemokines or enzymes that can degrade tumor stroma, thereby improving CAR-T cell infiltration and anti-tumor efficacy. This study utilized a CDX mouse model with HCC827 tumors, demonstrating that the combination of C8A8 antibody and IL-7-CAR-T cells significantly boosted the cytotoxic effect against HCC827 cells. Nevertheless, given the complex immunosuppressive environment and dense structure of solid tumors, additional validation with patient-derived tumor xenograft (PDX) models is required to thoroughly assess the combination therapy’s efficacy against NSCLC.

## Methods

### Research ethics

This study was approved by the Institutional Ethical Review Board at Peking Union Medical College Hospital (S-K1082) and the Institute of Zoology, Chinese Academy of Sciences (IOZ2016004). All procedures involving human participants adhered to the principles of the Declaration of Helsinki, with informed consent obtained from all participants.

### Culture of cell lines

Human peripheral blood mononuclear cells (PBMCs) were sourced from the Biobank of Peking Union Medical College Hospital. The cell lines used in this study included HEK293T, human lung adenocarcinoma cell lines A549 (ATCC, CCL-185), and HCC827 (ATCC, CRL-2868), all obtained from ATCC. These were cultured in Dulbecco’s Modified Eagle Medium (DMEM) supplemented with 10% fetal bovine serum, 2 mM GlutaMAX™-I, 1 mM sodium pyruvate, 1% MEM NEAA, and 1% penicillin/streptomycin at 37 °C in a 5% CO_2_ atmosphere. The human lung cancer line NCI-H23 (H23, ATCC, CRL-5800) and human erythroleukemia cell line K562 (ATCC, CCL-243) were also obtained from ATCC and cultured in RPMI-1640 media with 10% fetal bovine serum under the same conditions. Genes encoding a green fluorescent protein and firefly luciferase fusion protein (GFP/Luc) were transduced into K562, HCC827, and H23 cell lines for bioluminescence-based cytotoxicity assays.

### Animal study approval

All animal experiments were conducted in compliance with the policies and certification of the International Association for Assessment and Accreditation of Laboratory Animal Care (IOZ-YSB-2025-22). Animals were housed under specific pathogen-free (SPF) conditions at the animal facilities of the Institute of Zoology, Chinese Academy of Sciences. BALB/c mice were procured from SPF Biotechnology (Beijing), and 6-8-week-old male NOD-*Prkdc*^scid^  *IL-2rg*^null^ (NPG) mice were obtained from Vitalstar Biotechnology (Beijing).

### Purification of protein

The ex-PD-1 protein was expressed and purified using an insect cell expression system. Purification was performed using a His column (GE Biosciences) and an AKTA FPLC system. The purity of the protein was confirmed by SDS-polyacrylamide gel electrophoresis (SDS-PAGE).

### Production of monoclonal antibody (mAb)

To generate stable hybridoma cell lines, 8-week-old female BALB/c mice were subcutaneously injected with 100 μg of emulsified ex-hPD-1 protein or 1 × 10^7^ 3T3-hPD-1 cells. Hybridoma cells were prepared as previously described [50]. All mAbs were produced and purified from the hybridoma culture supernatant. The human–murine chimeric antibody, C8A8, was engineered by replacing the mouse IgG1 Fc region with the human IgG4 Fc region. Both C8A8 and Nivo were expressed and purified by Sino Biological, with all antibodies stored at −20°C until use.

### Enzyme-linked immunosorbent assay (ELISA)

Hybridoma cells were screened using indirect ELISA. High-binding capacity ELISA plates were coated with 100 µL of antigen (2 µg/mL) in coating buffer (Solarbio, C1055) and incubated overnight at 4 °C. The plates were then blocked with 5% skimmed milk in phosphate-buffered saline (PBS) containing 0.05% Tween-20 (PBST) at 37°C for 2 h. Hybridoma supernatants were added and incubated for 1 h at 37°C. After three washes, plates were incubated with horseradish peroxidase (HRP)-conjugated secondary antibody (Beyotime, A0201) diluted 1:3000 in blocking buffer at 37°C for 30 min. HRP activity was measured at 450 nm using a TMB Single-Component Substrate solution (Solarbio, PR1200), ELISA Stop Solution (Solarbio, C1058), and an ELISA plate reader (EL-808, Biotek). The blocking rate of anti-PD-1 mAbs was determined through ELISA. IL-7 cytokine secretion in the supernatants of NT, CAR-T, and IL-7-CAR-T cells was quantified using ELISA Kits (Multi-Science, EK107-96). Additionally, cytokine production (IFN-γ and IL-2) in the supernatants was analyzed using ELISA Kits (Multi-Science, EK102, EK180).

### Western blot

The 3T3-hPD-1 cells and 3T3 cells expressing empty vector (3T3-Vec) were lysed with RIPA lysis buffer. Cell lysates were mixed with loading buffer and separated on 10% SDS-PAGE gels. Proteins were transferred to a polyvinylidene difluoride (PVDF) membrane (Millipore). The membrane was blocked with 5% skimmed milk in Tris-buffered saline (TBS) containing 0.05% Tween-20 (TBST) at 37°C for 1 h, then incubated with antibody supernatants at 37°C for 1 h. An HRP-conjugated secondary antibody (Beyotime, A0201) diluted in TBST was applied to detect PD-1 protein, and immunoreactive bands were visualized using a Luminata Forte Western HRP Substrate Kit (Millipore, WBLUF0100).

### Immunofluorescence binding assays

Cells on gelatin-coated glass slides were fixed with 4% paraformaldehyde for 20 min, followed by washing with PBS and blocking with 5% bovine serum albumin (Sigma, A3912) for 1 h. Subsequently, the cells were stained overnight at 4°C with 5 μg/mL mAbs, followed by 1 h incubation at 37°C with Alexa Fluor 647 Goat anti-Mouse IgG (H + L) Cross-Adsorbed Secondary Antibody (Invitrogen, A-21236). DAPI (ZSGB-BIO, ZLI-9557) and DiO (Yeasen, 40725ES10) were used for counterstaining cell nuclei and membranes, respectively. Images were captured using a confocal laser scanning microscope.

### Surface plasmon resonance (SPR) measurements

SPR binding experiments were performed using a Biacore T100 instrument to determine mAb affinity. The mAbs were immobilized onto CM5 (Series S) sensor chips through amine coupling, and binding sensorgrams were analyzed using BiaEvaluation software.

### Lentivirus preparation

CAR construction followed established protocols [24]. The human IL-7 gene (Genebank, NM_000880.4) was synthesized by GenScript and subcloned into the lentiviral vector Fuw-EF1α-MCS. The human hPD-1 gene was inserted into the pCDH-CAG-P2A-mCherry lentiviral vector. The pLenti-CMV-EGFP-Luc-PGK-Puro lentiviral vector was purchased from OBiO Technology. Lentivirus production involved transfecting HEK293T cells with lentiviral vectors and packaging plasmids (psPAX2 and pMD2.G). Lentiviral supernatants were harvested and concentrated via ultracentrifugation at 48 h post-transfection.

### Generation of CAR-T cells

Fresh PBMCs were isolated using Ficoll-Paque PLUS (Cytiva, 17-1440-02) and sorted with a Pan T cell isolation kit (Miltenyi Biotec, 130-091-156). T cells were activated using CD3/CD28 Dynabeads (ThermoFisher, 11161D) and cultured in X-VIVOTM^15^ medium (Lonza, 04-418Q) supplemented with 5% fetal bovine serum, 2 mM GlutaMAX™-I, 1 mM sodium pyruvate, and 100 IU/mL recombinant Human IL-2 (PeproTech, 200-02). T cells were expanded and maintained at a density of 10^6^ cells/mL. After 36 h, T cells were washed and transduced with lentiviral supernatant.

### Flow cytometry

Cell surface expression was assessed using flow cytometry, with APC Anti-human EGFR (BioLegend, 352906) and APC anti-human PD-L1 (Biolegend, 329707) antibodies to detect EGFR and PD-L1 expression on tumor cells, respectively. Antibody incubations for flow cytometry were performed for 20 min at 4°C in PBS supplemented with 2% FBS. Apoptosis was evaluated using the AnnexinV-FITC/PI Apoptosis Detection Kit (YEASEN, 40303ES20). The binding affinity of mAbs was measured by incubating 1 μg mAb with 1 × 10^6^ 3T3-hPD-1 cells suspended in 100 μL PBS. Alexa Fluor Plus 488 Donkey anti-Mouse IgG (H + L) Highly Cross-Adsorbed Secondary Antibody (Invitrogen, A32766) or Alexa Fluor Plus 488 Donkey anti-human IgG (H + L) Highly Cross-Adsorbed Secondary Antibody (Invitrogen, A48276) was then added for detection.

### Cytotoxicity assay

CAR-T cell cytotoxicity was evaluated using a bioluminescence-based assay. Tumor-Luc cells (HCC827, H23, and K562) were seeded in triplicate at a density of 5000 cells per well in 96-well plates. Cells were co-cultured with non-transduced (NT), CAR-T, and IL-7-CAR-T cells at E: T ratios of 10:1, 3:1, and 1:1. To assess the impact of IL-7-CAR-T cell combination with PD-1 antibody on anti-tumor ability, cells were co-cultured with NT and IL-7-CAR-T cells at E: T ratios of 8:1, 4:1, and 2:1 in a medium containing anti-PD-1 antibody at a concentration of 30 μg/mL. Tumor cells were plated in 96-well plates at the same cell density. After 18 h, D-Luciferin sodium salt (Yeasen, 40901ES03) was added. Luminescence signals were measured using a PerkinElmer Victor X3 Reader. Specific lysis (%) = [(maximum luciferase activity − experimental luciferase activity)/maximum luciferase activity] × 100.

### Mouse study

Six to eight-week-old NPG mice were used for all xenograft experiments. All animal experiments were performed under the Institutional Animal Care and Use Committee guidelines. 4 × 10^5^ HCC827/Luc cells were subcutaneously injected into the right flank of 7-week-old male NPG mice. Four days post tumor xenograft, tumor size was assessed via bioluminescence imaging, and the mice were randomly divided into four groups. Mice were infused intravenously (i.v.) via the tail vein with 1 × 10^7^ NT, CAR-T, and IL-7-CAR-T cells. To evaluate the impact of the combination of IL-7-CAR-T cells with PD-1 antibody on anti-tumor efficacy *in vivo*, mice were infused intravenously via the tail vein with 5 × 10^6^ NT and IL-7-CAR-T cells after four days of tumor xenograft. 5 mg/kg C8A8 was administered intravenously on day 0, 3, 6, and 9. Isotype (Iso) and Nivo were utilized as negative and positive controls, respectively. Tumor progression was monitored every 7 days using the IVIS Spectrum Imaging platform.

### Histopathological and immunohistochemical analysis

To assess histopathological changes, tissues were collected from NPG mice 10 days post T cell infusion, fixed in 4% paraformaldehyde, and subjected to H&E staining. For IHC analysis, tissue sections were stained with anti-CD3 antibody (SerVice Bio, GB13014-50), followed by incubation with HRP-conjugated goat anti-rabbit IgG secondary antibody (SerVice Bio, GB23303). Microscopic evaluation of both H&E and IHC samples was performed using a BZ-X710 microscope (KEYENCE).

### Statistical analysis

All data were derived from at least three independent experiments and presented as mean ± S.D. Statistical analyses were performed using GraphPad Prism 8.0. Statistical significance was calculated using the unpaired Student’s *t*-test between the two groups. For comparison of survival curves, the log-rank test was performed. Statistical significance was indicated by asterisks and defined as *P < 0.05, **P < 0.01, ***P < 0.001, ****P < 0.0001.

## Supplementary Material

lnaf035_Supplementary_Data

## Data Availability

The data and materials during the current study are available from the corresponding author on reasonable request.
