# Supplementary material for: Integration of a novel anti-PD-1 antibody with chimeric antigen receptor-T engineered to express interleukin-7 enhances targeting efficacy against lung cancer
Source: Life Med. 2025 Dec 23;4(6):lnaf035. doi: 10.1093/lifemedi/lnaf035 (PMC12732667; doi:10.1093/lifemedi/lnaf035)
Supplement: lnaf035_Supplementary_Data [file lnaf035_supplementary_data.zip › 3.docx]

## Integration of a Novel Anti-PD-1 Antibody with CAR-T Engineered to Express IL-7 Enhances Targeting Efficacy against Lung Cancer

Chenxi Cheng^1,2,3^, Lin Zhang^1,2,3^, Jiani Cao^1,2^, Xiaoyan Li^1,2^,

Ya Wen^1,2,3^, Kun Liu^1,2,3,^*, Tongbiao Zhao^1,2,3^^,^*

^1^Key Laboratory of Organ Regeneration and Reconstruction, State Key Laboratory of Stem Cell and Reproductive Biology, Institute for Stem Cell and Regeneration, Institute of Zoology, Chinese Academy of Sciences, Beijing 100101, China

^2^University of Chinese Academy of Sciences, Beijing 100049, China

^3^Beijing Institute for Stem Cell and Regenerative Medicine, Beijing 100101, China

*Correspondence: 87liukun@163.com (K.L.), tbzhao@ioz.ac.cn (T.Z.)

**Supplementary figure legends**

## Supplementary Figure 1. Sera from mice immunized with ex-hPD-1 proteins and ex-hPD-1-expressing 3T3 cells contain anti-PD-1 antibodies.

### (A) SDS-PAGE analysis of ex-hPD1 protein expression and purification using an insect cell expression system. (B) Measurement of human PD-1 overexpression on the surface of 3T3 cells and A549 cells by flow cytometry. (C) Recognition of human PD-1 on the surface of A549 cells by serological antibodies obtained from mice immunized with ex-hPD-1 protein or 3T3-hPD-1 cells. (D) As assessed by ELISA, serum from mice immunized with ex-hPD-1 protein or 3T3-hPD-1 cells specifically recognized the ex-PD-1 protein. Data shown are the mean ± SD, *n* = 3; *****P* < 0.0001, Student’s *t*-test.

**Supplementary Figure 2. Both EGFR and PD-L1 are highly expressed in lung cancer cells.**

(A) Surface expression of EGFR on lung cancer cells (HCC827, H23), with K562 cells used as a negative control. (B) Surface expression of PD-L1 on lung cancer cells (HCC827, H23), with K562 cells used as a control.

**Supplementary Figure 3. IL-7 endows CAR-T cells with resistance to cancer cell-induced apoptosis.**

(A) Schematic diagram depicting the CAR structure. (B) Flow cytometry analysis expression of CAR and IL-7-CAR on T cells. (C) Quantitative analysis of IL-7 mRNA expression levels in CAR-T and IL-7-CAR-T cells. Data shown are the mean ± SD, *n* = 4; *****P* < 0.0001, Student’s *t*-test. (D) IL-7 inhibits apoptosis of CAR-T cells. Apoptosis of NT, CAR-T, and IL-7-CAR-T cells was measured by flow cytometry after co-culture with A549 cells. (E) Statistical analysis of cellular apoptosis. Data are shown as the mean ± SD, *n* = 4; **P* < 0.05, Student’s *t*-test. (F) The expression of PD-1, Tim3, and LAG-3 on CAR-T cells and IL-7-CAR-T cells was analyzed by FACS. Data shown are the mean ± SD, *n* = 3; ***P* < 0.01, Student’s *t*-test.

**Supplementary Figure 4. IL-7-engineered CAR-T cells exhibit robust *in vivo* safety with minimal off-target toxicity.**

(A) Tissues were harvested from NPG mice 10 days post T cell infusion and subjected to hematoxylin and eosin (H&E) staining. (B) Immunohistochemical staining of tissues using anti-CD3 antibody.

**Supplementary Figure 5. Combination therapy of IL-7-CAR-T cells and anti-PD-1 antibody maintains favorable safety and demonstrates enhanced pharmacokinetic properties.**

(A) Tissue samples were harvested from NPG mice at day 10 post-adoptive transfer, following combination therapy with IL-7-CAR-T cells and anti-PD-1 antibody. All tissues were stained with H&E. (B) Balb/c mice (*n* = 4 biologically independent animals) were administered 5 mg/kg C8A8 or Nivo via intravenous injection. Serum concentrations of C8A8 and Nivo were determined by ELISA at the specified time points.
