# Supplementary material for: Integration of a novel anti-PD-1 antibody with chimeric antigen receptor-T engineered to express interleukin-7 enhances targeting efficacy against lung cancer
Source: Life Med. 2025 Dec 23;4(6):lnaf035. doi: 10.1093/lifemedi/lnaf035 (PMC12732667; doi:10.1093/lifemedi/lnaf035)

Supplementary Figure 1 Sera from mice immunized with ex-hPD-1 proteins or ex-hPD-1-expressing 3T3 cells contain anti-PD-1 antibodies

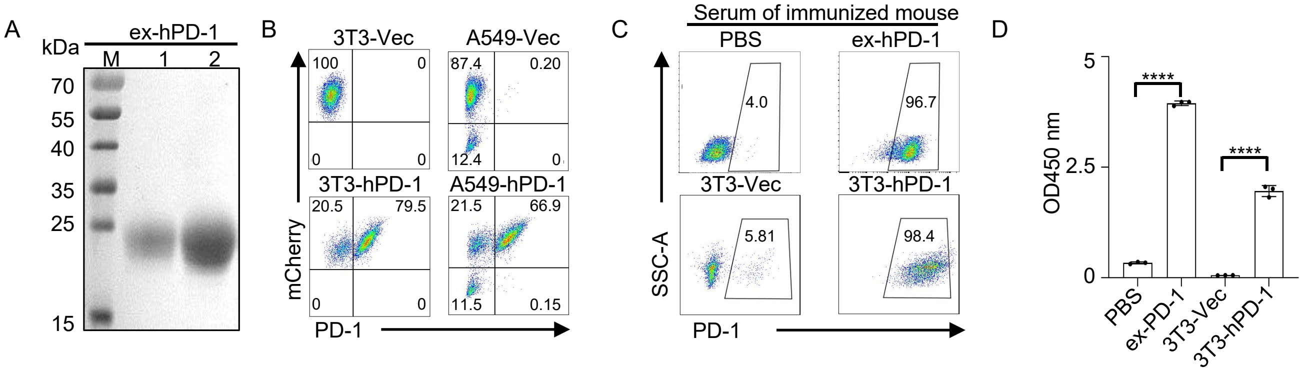

Supplementary Figure 2 Both EGFR and PD-L1 are highly expressed in lung cancer cells

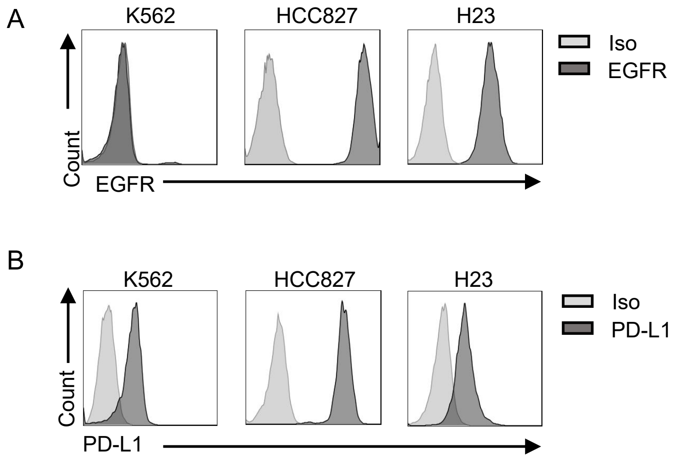

Supplementary Figure 3 IL-7 endows CAR-T cells with resistance to cancer cell-induced apopto

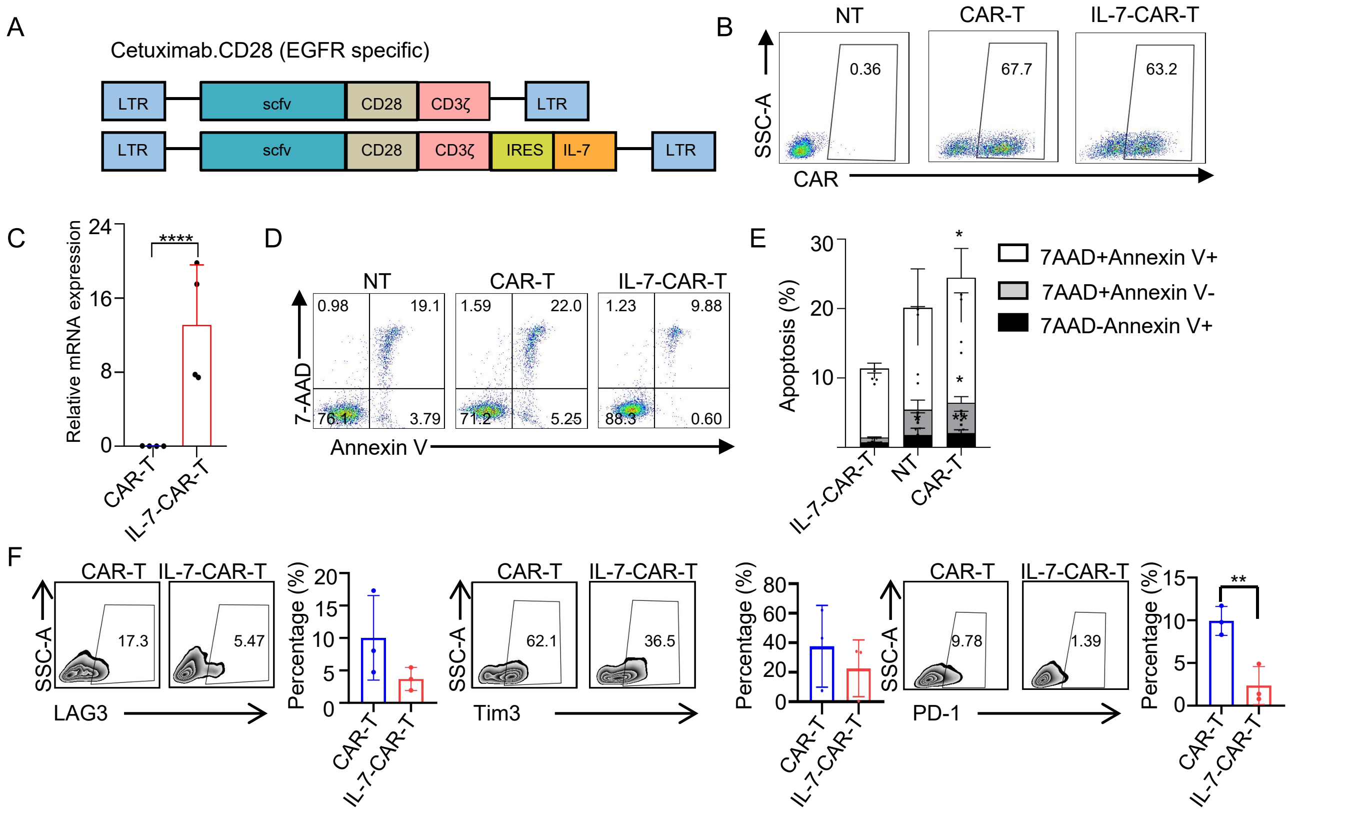

Supplementary Figure 4 IL-7-engineered CAR-T cells exhibit robust in vivo safety with minimal off-target toxicity

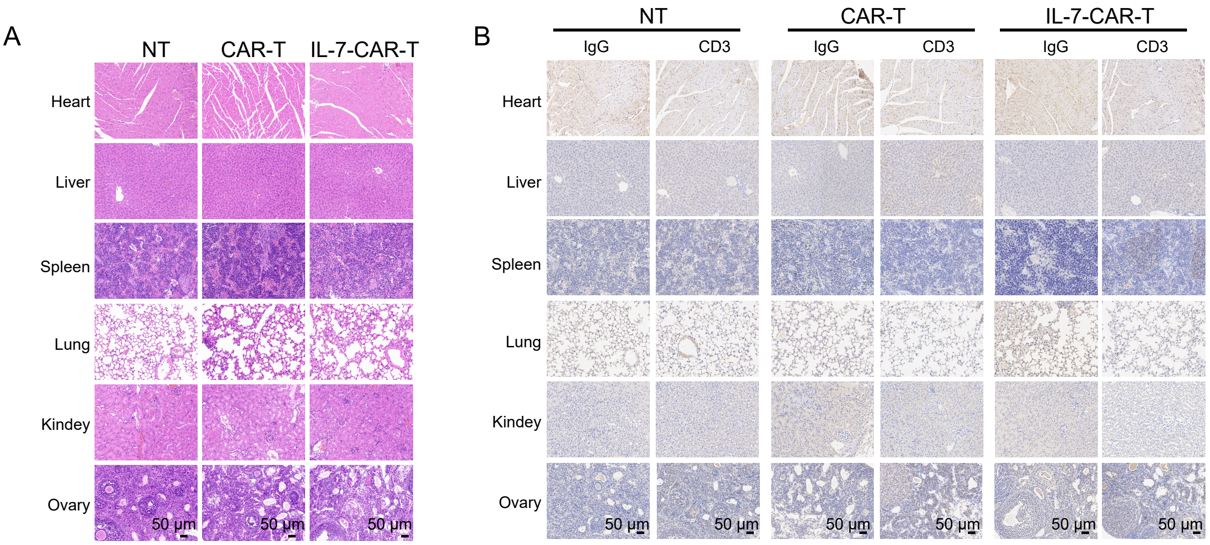

Supplementary Figure5 Combination therapy of IL-7-CAR-T cells and anti-PD-1 antibody maintains favorable safety and demonstrates enhanced pharmacokinetic properties

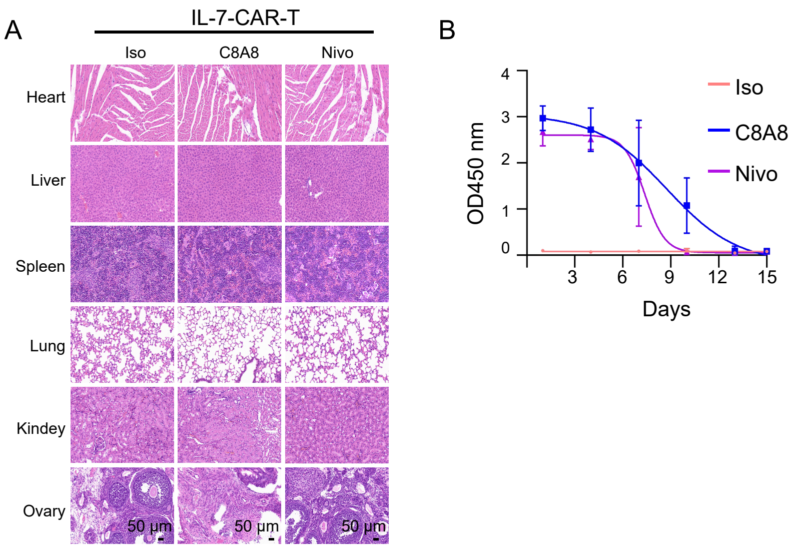

Supplement: lnaf035_Supplementary_Data [file lnaf035_supplementary_data.zip › Integration of a Novel Anti-PD-1 Antibody with CA_SI figures.pdf]
